# Supplementary material for: Pyrethroid Resistance Situation across Different Eco-Epidemiological Settings in Cameroon
Source: Molecules. 2022 Sep 26;27(19):6343. doi: 10.3390/molecules27196343 (PMC9573433; doi:10.3390/molecules27196343)
Supplement: Supplementary file 1 [file molecules-27-06343-s001.zip › molecules-1897028-supplementary.pdf]

Table S1 : Susceptibility test of *Anopheles gambiae* s.l. with permethrin.

	Jul-Aug 2019				Jul -Aug 2020				Jul-Aug 2021			
	Perm 1X	Perm 5X	Perm 10X	PBO+Perm	Perm 1X	Perm 5X	Perm 10X	PBO+Perm	Perm 1X	Perm 5X	Perm 10X	PBO+Perm
	N(%)	N(%)	N(%)	N(%)	N(%)	N(%)	N(%)	N(%)	N(%)	N(%)	N(%)	N(%)
**Santchou**	80(2)	100(19)	100(38)	100(27)	100(2)	100(17)	100(30)	100(30)	100(15)	100(20)	100(25)	100(15)
**Bertoua**	100(11)	100(68)	96(95)	100(13)	100(9)	100(66)	100(95)	100(11)	83(5.6)	86(76.74)	80(96.25)	80(71.25)
**Tibati**					100(0)	80(35)	80(97.5)	100(11)	(4.80)	(65.04)	(94.26)	100(69)
**Kaélé**					92(19.56)	82(53.66)	84(66.67)	100(42)	96(4)	84(82)	83(62)	98(51)
**Njombé**									100(52)	100(97)	100(99)	100(89)
**Kékem**									80(1)	80(20)	80(75)	80(3)
**Bélabo**									88(0)	97(48.45)	100(93)	93(38.71)

Table S2: Susceptibility test of *Anopheles gambiae* s.l. with deltamethrin

	Jul-Aug 2019				Jul -Aug 2020				Jul-Aug 2021			
	Delta 1X	Delta 5X	Delta 10X	PBO+Delta	Delta 1X	Delta 5X	Delta 10X	PBO+Delta	Delta 1X	Delta 5X	Delta 10X	PBO+Delta
	N(%)	N(%)	N(%)	N(%)	N(%)	N(%)	N(%)	N(%)	N(%)	N(%)	N(%)	N(%)
**Santchou**	100(2)	100(32)	100(21)	100(38)	100(4)	100(25)	100(21)	100(40)	100(20)	100(50)	80(47.5)	80(33.75)
**Bertoua**	100(49)	100(69)	100(99)	100(100)	100(50)	100(69)	100(95)	100(31)	80(63.41)	80(96.25)	80(86.25)	80(96.25)
**Tibati**					82(41.46)	100(59)	80(72.5)	100(69.41)	100(41)	(71.43)	(71.42)	100(82)
**Kaélé**					96(22.92)	84(28.57)	83(33.73)	98(58.16)	92(8)	82(81)	84(57)	100(100)
**Njombé**									100(91)	100(96)	100(99)	100(100)
**Kékem**									80(4)	80(21)	80(75)	80(100)
**Bélabo**									92(51.96)	92(61.96)	97(72.16)	88(61.36)
